# Healthy low nitrogen footprint diets

**DOI:** 10.1016/j.gfs.2019.100342

**Published:** 2020-03

**Authors:** João Costa Leite, Sandra Caldeira, Bernhard Watzl, Jan Wollgast

**Affiliations:** a Faculdade de Ciências da Saúde, Universidade Fernando Pessoa, Porto, Portugal; b Center for Health Technology and Services Research (CINTESIS), Porto, Portugal; c European Commission, Joint Research Centre (JRC), Ispra, Italy; d Department of Physiology and Biochemistry of Nutrition, Max Rubner-Institut, Karlsruhe, Germany

**Keywords:** Vegetarian diet, Nitrogen footprint, Health, Food-based dietary guidelines, Food systems

## Abstract

Shifting towards more plant-based diets can reduce the environmental burden of the food system including its impact on the nitrogen cycle. However, such changes need to be compatible with healthy nutrition. To discuss the health aspects of plant-based dietary patterns, this literature review analyses vegetarian and vegan diets and concludes that well-planned, balanced vegetarian diets are nutritious and healthy. Food-based dietary guidelines (FBDGs) that include environmental aspects and practical advice to individuals and society are needed as crucial instruments to further promote public health within the planetary boundaries. FBDGs need to be better exploited to serve as a basis to policies that promote diets supporting the UN sustainable development goals.

## Highlights

• Balanced vegetarian diets are nutritious, healthy and have low nitrogen footprints.
• Restricting animal foods from diet needs to be managed appropriately.
• An interconnected system of factors and actors affects food consumption.
• Policies promoting healthy low footprint diets effectively are needed.
• Updated dietary recommendations can provide guidance to individuals and policy.

## Graphical abstract

**Figure:**
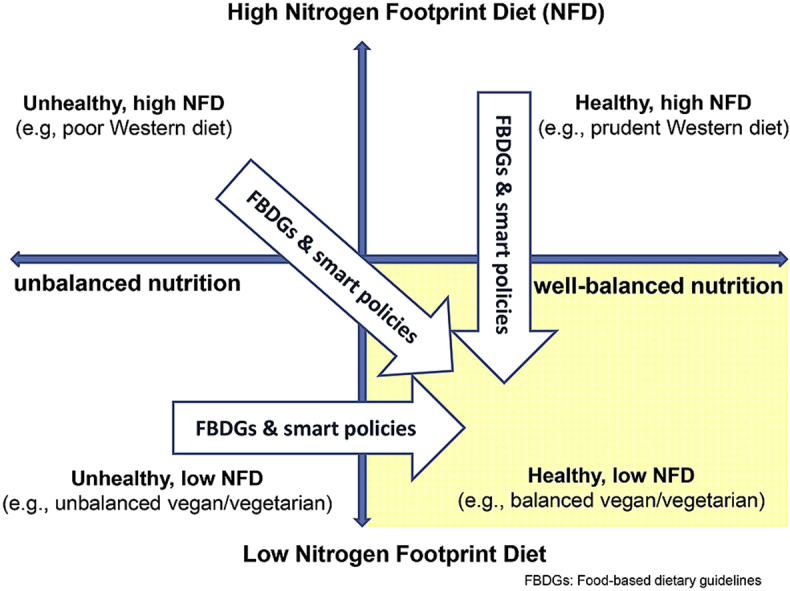

## 1 Introduction

Current food systems have a major impact on the environment. They may also result in poor diets driving the global burden of obesity and other non-communicable diseases, while still leaving as many people in food insecurity and hunger (Tilman and Clark, 2014; Godfray et al., 2018). Scaling up healthy and environmentally sustainable diets for everyone is an important goal and one way to achieve it is to substantially reduce the consumption of animal-sourced food products and promote plant-based diets (Westhoek et al., 2015; Willett et al., 2019). Compared to plant-based foods, animal-sourced foods and in particular red meat require higher input of resources, mainly linked to animal feed, and have higher impacts on the environment, including on the nitrogen cycle. Nitrogen is an essential nutrient for plant growth, key to agricultural productivity. The application of nitrogen and phosphorous fertilisers is a regular practice to ensure productivity and food availability to feed an increasingly populated planet (Galloway et al., 2014; Steffen et al., 2015). However, a significant proportion of the nitrogen applied to soils is lost to the environment mostly through volatilization and leaching. Globally, reactive nitrogen emissions due to human activity are already beyond the planet's safe operating space and are a main cause for soil acidification, water and air quality deterioration and climate change (Sutton et al., 2011; Leip et al., 2015; Steffen et al., 2015). Reducing the intake of animal-based foods at population level and shifting towards more plant-based diets, such as vegetarian diets, are potential approaches to manage nitrogen emissions within planetary boundaries (Springmann et al., 2018a) and carry other environmental benefits as well. Whilst animal-sourced foods also have considerably higher water footprints, greenhouse gas (GHG) footprints and land use than plant-based foods we focus on nitrogen as a proxy for the environmental impact of the food system (Mekonnen and Hoekstra, 2012; Weiss and Leip, 2012; Poore and Nemecek, 2018).

The nitrogen footprint is the sum of all nitrogen losses to the environment from the supply chain of a food – from the production of input required on the farm through the processing and trading, consumption and end-of life (Leach et al., 2012; FAO, 2018; Leip and Uwizeye, 2019). The majority of nitrogen emissions occur at the farm, and some authors provide N footprint data only up to the farm gate. For the European Union, the cradle-to-farmgate N footprint of meat was estimated in the range between 50 and several 100 g N (kg product)^−1^, while most vegetables products are in the range 1–10 g N (kg product)^−1^ (Leip et al. 2014, 2015).

There are dietary patterns such as the Mediterranean or the Nordic diet that include animal sourced foods and are practical examples of nutritionally adequate, healthful and rather sustainable diets with low N footprints. This perspective however focuses on vegetarian diets, defined here as all dietary patterns that completely exclude meat and fish. These have considerably lower nitrogen footprints (Scarborough et al., 2014; van Dooren et al., 2014; Castañé and Antón, 2017; Dagnelie and Mariotti, 2017; Springmann et al., 2018b) while vegan diets, through the exclusion of all animal-derived foods, have the lowest nitrogen footprint (Scarborough et al., 2014; Springmann et al., 2018b). Excluding meat, fish, milk, eggs and all animal-derived foods from diets can however, put individuals at risk for nutritional inadequacies and may be particularly challenging in certain socio-cultural or economic contexts. For instance, in several low-income countries, societies rely on animal products to provide nutrition security to individuals that have limited access to a diverse and nutritious rich plant based diet (Godfray et al., 2018; Springmann et al., 2018a). On the other hand, reducing meat and protein intake in high income countries, where meat consumption is above the levels recommended and protein intake above those required, can provide both health gains and reduce environmental impacts from food consumption (Springmann et al., 2018a). Whilst an estimated 1.5 billion individuals in the world are vegetarians ‘by necessity’ and strive to consume meat as soon as they can afford it, a much smaller but increasing number of individuals are vegetarians by choice, generally living in high income countries (Leahy et al., 2010). Motives include ethics, religion, health and environmental concerns and estimates of vegetarians vary greatly between less than 1% to more than 10% of populations in high-income countries (Leitzmann and Keller, 2013; Janssen et al., 2016; Richter et al., 2016; Agnoli et al., 2017; Corrin and Papadopoulos, 2017). Reducing food waste and changing diets, in particular reducing meat consumption, are increasingly seen as necessary in high income populations to meet food demands for a growing world population (German Federal Ministry of Food and Agriculture, 2019). This perspective compares health-related outcomes between vegetarian, vegan and omnivorous diets and discusses both challenges and opportunities of the lower nitrogen footprint diets. This is done through the lenses of high-income countries, where most individuals can have plenty of choice. In addition, we explore possible strategies to facilitate and promote the adoption of all forms of low nitrogen footprint diets in a nutritionally adequate and healthful manner and highlight the importance of food based dietary guidelines (FBDGs) in this context.

## 2 Methods

A literature search using Google Scholar and Pubmed was conducted to summarise the impact of vegetarian diets on health outcomes by extracting data on the estimated effects from meta-analyses of cohort studies and randomised clinical trials comparing vegetarian and omnivorous diets. Meta-analyses of cross-sectional studies were not considered as these provide lower quality of evidence. Meta-analyses of cohort studies provided data on the relative effect of vegetarian diets compared to omnivorous diets on chronic disease progression and were searched by using the search terms ‘vegetarian’, ‘vegan’, ‘meta-analysis', ‘cohort’ combined with relevant health outcomes including ‘cardiovascular disease’, 'diabetes', ‘cancer’, ‘bone health’ or ‘mortality’. Meta-analyses of randomised clinical trials were searched to extract data on the impact of vegetarian diets on health risk factors. The search terms included ‘vegetarian’, ‘vegan’, ‘meta-analysis', ‘clinical trials', combined with risk factors including ‘blood lipids', ‘cholesterol’, ‘glucose’, ‘insulin’, ‘blood pressure’, ‘weight loss', ‘body weight’ or ‘inflammatory markers'. Vegan diets were treated separately from vegetarian diets whenever specific data was available.

To provide an overview of the existing official positions and guidelines regarding vegetarian diets, position statements from scientific societies were searched in Google Scholar, Pubmed and Google search engine using combined terms including ‘statement’, ‘scientific position’, dietary guidelines', ‘vegetarian’, ‘vegan’, ‘scientific society’ complemented by terms that identified internationally recognized scientific societies in the field of food and health and their own websites. Position statements were extracted and compiled from identified documents. Limited to available online data and the English language, healthy eating guidelines for vegetarians from national governmental bodies were searched by combining ‘dietary guidelines', ‘vegetarian’, ‘vegan’ and ‘food based dietary guidelines' The FAO food based dietary guidelines platform and the European Commission Health Promotion and Disease Prevention Knowledge Gateway were accessed to double check available data (European Commission, 2019a; FAO, 2019).

### 2.1 Comparison of health-related outcomes between vegetarian, vegan and omnivorous diets

A total of 13 meta-analyses (published between 2009 and 2019) of cohort studies (n = 6) and randomized controlled trials (n = 7) were identified and listed in Table 1.

**Table 1:** Health impact of vegetarian diets in comparison to omnivorous diets: summary of meta-analyses of cohort studies and randomised controlled trials.

Study	Title	Year	Outcomes	Studies	N	Effect (vegetarian vs omnivorous)^a^^,^^b^
Dinu et al.	Vegetarian, vegan diets and multiple health outcomes: A systematic review with meta-analysis of observational studies	2016	Multiple health outcomes	n =10 cohort studies	72,298	IHD incidence and/or mortality: RR = 0.75 (95% CI, 0.68–0.82); Incidence total cancer: vegetarians RR = 0.92 (95% CI,0.87–0.98) vegans RR = 0.85 (95% CI, 0.75–0.95); All-cause mortality: vegetarians RR = 0.94^ns^ (95% CI, 0.86–1.04) vegans RR = 0.88^ns^ (95% CI, 0.75-1.02); Total cardiovascular disease incidence/mortality: RR = 0.93^ns^ (95% CI, 0.86–1.00); Cerebrovascular disease incidence/mortality: RR = 0.93^ns^ (95% CI, 0.78–1.10); Breast cancer incidence: RR 0.94^ns^ (95% CI, 0.84–1.06); Colorectal mortality: RR = 0.90^ns^ (95% CI, 0.76–1.05); Breast cancer mortality: RR = 0.94^ns^ (95% CI, 0.56–1.58); Prostate cancer mortality: RR = 0.90^ns^ (95% CI, 0.63–1.29); Lung cancer mortality: RR = 0.86^ns^ (95% CI, 0.62–1.19)
Godos et al.	Vegetarianism and breast, colorectal and prostate cancer risk: an overview and meta-analysis of cohort studies	2016	Prostate cancer, breast cancer, colo-rectal cancer	n = 6 cohort studies	686,629	Breast cancer: RR = 0.96^ns^ (95% CI, 0.88–1.05); Colorectal cancer: RR = 0.88^ns^ (95% CI, 0.74–1.05); Prostate cancer: RR = 0.83^ns^ (95% CI, 0.63–1.10)
Huang et al.	Cardiovascular Disease Mortality and Cancer Incidence in Vegetarians: A Meta-Analysis and Systematic Review	2012	Cardiovascular Disease Mortality and Cancer Incidence	n = 7 cohort studies	124,706	IHD mortality: RR = 0.71; (95% CI, 0.56–0.87) Cancer incidence: RR = 0.82; (95% CI, 0.67–0.97)
Iguacel et al.	Veganism, vegetarianism, bone mineral density, and fracture risk: a systematic review and meta-analysis	2019	Bone Health	n = 19 cohort studies	37 134	Lumbar spine BMD MD: 0.032; (95% CI, - 0.048 to - 0.015); subgroup vegans MD, - 0.070; (95% CI, - 0.116 to - 0.025), vegetarians MD: 0.023; (95% CI, 0.035 to 0.010) Femoral neck BMD MD: 0.037; (95% CI, - 0.054 to −0.020), subgroup: vegetarians MD: 0.025; (95% CI, - 0.038 to - 0.012), vegans MD, - 0.055; (95% CI, - 0.090 to - 0.021); Whole body BMD MD: 0.048; (95% CI, - 0.080 to - 0.016); subgroup vegans MD: 0.059; (95% CI, 0.106 to 0.012): vegetarians MD: 0.035^ns^; (95% CI, 0.093 to 0.023); Fracture risk RR: 1.316; (95% CI, 1.038–1.668); Subgroup vegans RR 1.439; (95% CI, 1.047–1.977); vegetarians RR: 1.254^ns^; (95% CI, 0.917–1.714)
Kwok et al.	Vegetarian diet, Seventh Day Adventists and risk of cardiovascular mortality: A systematic review and meta-analysis	2014	Risk cardiovascular mortality	n = 8 cohort studies	183,321	Adventists vs non-Adventists mortality risk: RR = 0.68 (95% CI, 0.45–1.02) vs RR = 1.04 ^ns^ (95% CI, 0.98–1.10) IHD: RR = 0.60 (95% CI, 0.43–0.80) vs RR = 0.84 (95% CI, 0.74–0.96) Cerebrovascular disease: RR = 0.71 (95% CI, 0.41–1.20) vs RR = 1.05 ^ns^ (95% CI, 0.89–1.24)
Lee et al.	Adherence to a Vegetarian Diet and Diabetes Risk: A Systematic Review and Meta-Analysis of Observational Studies	2017	Diabetes risk	n = 14; 2 cohort 12 cross sectional	not reported	Cohorts: OR = 0.64 (95% CI, 0.57–0.74); Cohorts and Cross-Sectional: vegetarians OR = 0.73 (95% CI, 0.61–0.87); vegans OR = 0.59 (95% CI, 0.39–0.91)
Barnard et al.	A Systematic Review and Meta-Analysis ofChanges in Body Weight in Clinical Trials of Vegetarian Diets	2015	Weight reduction	n = 4 RCTs	453	Intention-to-treat analysis MD: 3.4 kg (95% CI, -4.4 to -2.4) Completer analysis MD: 4.6 kg (95% CI, -5.4 to -3.8)
Huang et al.	Vegetarian Diets and Weight Reduction: a Meta-Analysis of Randomized Controlled Trials	2015	Weight reduction	n = 12 RCTs	1151	MD: −2.02 kg (95% CI, -2.80 to -1.23)
Viguiliouk et al.	Effect of vegetarian dietary patterns on cardiometabolic risk factors in diabetes: A systematic review and meta-analysis of randomized controlled trials	2018	Cardiometabolic risk factors in diabetes	n = 9 RCTs	664	HbA1c (n = 8) MD: 0.29% (95% CI, -0.45 to -0.12) Glucose MD (n = 6): -0.56 mmol/L (95% CI, -0.99 to −0.13) LDL-C MD (n = 6): -0.12 mmol/L (95% CI, -0.20 to −0.04) Non-HDL-C MD (n = 7): -0.13 mmol/L (95% CI,-0.26 to - 0.01) HDL-C (n = 8): 0.03 mmol/L ^ns^ (95% CI, 0.08, 0.02) Body weight MD (n = 6): 2.15 kg (95% CI, -2.95 to-1.34 kg) Triglycerides MD (n = 7): 0.14 mmol/L^ns^ (95% CI, -0.10 to 0.38) SBP MD (n = 7): 0.10 mmHg^ns^ (95% CI, -2.33 to 2.52) DBP MD (n = 7): 0.53 mmHg^ns^ (95% CI,-0.50 to 1.57)
Wang et al.	Effects of Vegetarian Diets on Blood Lipids: A Systematic Review and Meta-Analysis of Randomized Controlled Trials	2015	Blood lipids	n = 11 RCTs	832	Total cholesterol MD: -0.36 mmol/L (95% CI, -0.55 to - 0.17) LDL-C MD: -0.34 mmol/L (95% CI, -0.57 to - 0.11) HDL-C MD: -0.10 mmol/L (95% CI, -0.14 to -0.06) Non–HDL-C MD: -0.30 mmol/L (95% CI, -0.50 to -0.10) Triglycerides MD: 0.04 mmol/L^ns^ (95% CI, -0.05 to 0.13)
Yokoyama et al.	Association between plant-based diets and plasma lipids: a systematic review and meta-analysis	2017	Blood lipids	n = 19 RCTs	1484	Total cholesterol MD: -12.5 mg/dL (95% CI, - 17,8 to −7,2) LDL-C MD: -12.2 mg/dL (95% CI, -17,7 to -6,7) HDL-C MD: -3.4 mg/dL (95% CI, -4,3 to -2,5) Triglycerides MD: 5.8^ns^ mg/dL (95% CI, -0.9 to 12,6)
Yokoyama et al.	Vegetarian diets and glycaemic control in diabetes: a systematic review and meta-analysis	2014	Glycaemic control in diabetes	n = 6 RCTs	255	HbA1c MD: -0.3% (95% CI, --0.62 to -0.15) Glucose MD: -0.36 mmol/L (95% CI, -1.04 to -0.32)
Yokoyama et al.	Vegetarian Diets and Blood Pressure A Meta-analysis	2014	Blood pressure	n = 7 RCTs	311	SBP MD: -4.8 mm Hg (95% CI, -6.6 to -3.1) DBP MD: -2.2 mm Hg (95% CI: -3.5 to -1.0)

N, number of pooled individuals; MD, mean difference; CI, confidence interval; RR, relative risk; OR, odds ratio; IHD, ischemic heart disease; BMD, bone mineral density; LDL-C, low-density lipoprotein cholesterol; HDL-C, high-density lipoprotein cholesterol; SBP, systolic blood pressure; DBP, diastolic blood pressure.

a All effect differences are between total vegetarian and omnivorous populations except when described.

b All effects are significantly difference except when described; ^ns^ non-significant.

The combined analysis of these studies supports a positive effect of vegetarian relative to omnivorous diets in preventing chronic disease by reducing the risks for ischemic heart disease (relative risk (RR) = 0.75; 95% CI, 0.68–0.82), diabetes (OR = 0.64; 95% CI, 0.57–0.74) and total cancer incidence (RR = 0.92; 95% CI, 0.87–0.98) (Huang et al., 2012; Dinu et al., 2017; Godos et al., 2017; Lee and Park, 2017). Vegan diets are similarly associated with lower risks of developing diabetes (RR = 0.59; 95% CI, 0.39–0.91) and a reduced cancer incidence (RR = 0.85; 95% CI, 0.75–0.95) compared with non-vegetarians (Kwok et al., 2014; Lee and Park, 2017). These effects are likely due to a positive impact of vegetarian diets on body weight, blood pressure, blood lipids, glycaemic control as supported by existing systematic reviews of clinical trials (Yokoyama et al. 2014a, 2014b, 2017; Barnard et al., 2015; Wang et al., 2015; Huang et al., 2016; Viguiliouk et al., 2018). However, bone health concerns appear stronger among vegans with a significant increased risk for fractures (RR = 1.44, 95%CI 1.05–1.98) (Kwok et al., 2014; Lee and Park, 2017; Iguacel et al., 2019). The above studies note some relevant limitations that may influence the reported outcomes. For instance, most of the meta-analyses included a small number of studies only, reflecting the scarcity of vegetarian cohorts. The results obtained with the Adventists and non-Adventists cohorts also show the importance of adjusting for confounders including body weight, alcohol consumption, physical activity and smoking. Other aspects that may contribute to heterogeneity across studies are different definitions of vegetarianism, its duration and follow-up periods. In addition, only a few studies reported sub-group comparisons (for example vegans and ovo-lacto-vegetarians) which could be relevant to explore health outcome differences within vegetarian diets. Due to the limited number of studies and sample sizes, vegan diets outcomes need to be interpreted cautiously. Different food intake assessment methods may also confound the results (Alles et al., 2017; Segovia-Siapco and Sabate, 2018). Excluding animal-sourced foods from the diet has often been linked to increased risks for inadequate intakes of some critical micronutrients such as vitamin B12, zinc, iron or iodine (Foster et al., 2013; Obersby et al., 2013; Pawlak et al., 2014; Woo et al., 2014; Sobiecki et al., 2016; Alles et al., 2017; Haider et al., 2018). Plant-based diets that favour fruit juices, refined grains, sweets, desserts and sugar-sweetened beverages have even been associated with increased coronary heart disease risk confirming that vegetarian diets are not necessarily healthy (Satija et al., 2017). Whilst existing evidence supports the potential role of vegetarian diets in disease prevention, as in all diets, principles of healthy and nutritionally balanced eating must be applied to reap their full potential.

### 2.2 Position statements and dietary guidelines related to vegetarian diets

Position statements and dietary guidelines from scientific and governmental institutions are important sources of healthy eating recommendations. All EU member states have issued food-based dietary guidelines (FBDGs) to support their citizens and guide their food-related policies (European Commission, 2019a). Concrete position statements and guidance for vegetarian or vegan diets are however less common. Table 2 summarises the position statements published by seven scientific societies on vegetarian and vegan diets (American Dietetic Association and Dietitians of Canada, 2003; Amit et al., 2010; Garton, 2016; Melina et al., 2016; Richter et al., 2016; Agnoli et al., 2017; Fewtrell et al., 2017).

**Table 2:** Official positions and statements from scientific and governmental bodies on vegetarian diets.

Country	Year	Body	Title	Position/Statements
Italy	2017	Italian Society of Human Nutrition	Position paper on vegetarian diets from the working group of the Italian Society of Human Nutrition	“Well-planned vegetarian diets that include a wide variety of plant foods, and a reliable source of vitamin B12, provide adequate nutrient intake. Government agencies and health/nutrition organizations should provide more educational resources to help Italians consume nutritionally adequate vegetarian diets."
UK	2017	British Dietetic Association	Food Fact Sheet: Plant Based Diet and Vegetarian Diets	“Well-planned vegetarian diets are appropriate for all stages of life and have many benefits. These guidelines will help you enjoy all the health benefits and ensure you're eating a nutritious and complete diet. The government's eatwell plate still applies to vegetarians"
Europe	2017	European Society for Paediatric Gastroenterology, Hepatology, and Nutrition (ESPGHAN) Committee on Nutrition	Complementary Feeding: A Position Paper by the European Society for Paediatric Gastroenterology, Hepatology, and Nutrition (ESPGHAN) Committee on Nutrition	“Care is required to ensure an adequate nutrient intake during complementary feeding when vegetarian or vegan diets are used. Although theoretically a vegan diet can meet nutrient requirements the risks of failing to follow advice are severe [ …] If a parent chooses to wean an infant onto a vegan diet this should be done under regular medical and expert dietetic supervision …”
USA	2016	Academy of Nutrition and Dietetics	Position of the Academy of Nutrition and Dietetics: Vegetarian Diets	“It is the position of the Academy of Nutrition and Dietetics that appropriately planned vegetarian, including vegan, diets are healthful, nutritionally adequate, and may provide health benefits in the prevention and treatment of certain diseases. These diets are appropriate for all stages of the life cycle, including pregnancy, lactation, infancy, childhood, adolescence, older adulthood, and for athletes."
Germany	2016	German Nutrition Society	Vegan Diet - Position of the German Nutrition Society (DGE)	“The DGE does not recommend a vegan diet for pregnant women, lactating women, infants, children or adolescents. Persons who nevertheless wish to follow a vegan diet should permanently take a vitamin B12 supplement, pay attention to an adequate intake of nutrients, especially critical nutrients, and possibly use fortified foods or dietary supplements. They should receive advice from a nutrition counsellor and their supply of critical nutrients should be regularly checked by a physician."
Canada	2010	Canadian Paediatric Society	Vegetarian diets in children and adolescents	“A well-balanced vegetarian diet can provide for the needs of children and adolescents. However, appropriate caloric intake should be ensured and growth monitored. Particular attention should be paid to adequate protein intake and sources of essential fatty acids, iron, zinc, calcium and vitamins B12 and D. Supplementation may be required in cases of strict vegetarian diets with no intake of any animal products."
Canada	2003	Dietitians of Canada	Position of the American Dietetic Association and Dietitians of Canada: Vegetarian diets	“Appropriately planned vegetarian diets are healthful, nutritionally adequate, and provide health benefits in the prevention and treatment of certain diseases. Well-planned vegan and other types of vegetarian diets are appropriate for all stages of the life cycle, including during pregnancy, lactation, infancy, childhood, and adolescence."

Five of these position statements highlight that well-planned vegetarian diets can be nutritionally adequate. The Academy of Nutrition and Dietetics, Dietitians of Canada and the British Dietetic Association go further and highlight that well-planned balanced vegetarian diets are appropriate across all stages of life. However, the German Nutrition Society does not recommend a vegan diet for pregnant women, infants, children and adolescents. The European Society for Paediatric Gastroenterology (ESPGHAN) also highlights that during weaning vegan diets should only be used under appropriate supervision.

Importantly, the Italian Society of Human Nutrition points out that ‘government agencies and health/nutrition organizations should provide more educational resources to help Italians consume nutritionally adequate vegetarian diets' (Agnoli et al., 2017). Indeed, our own search, albeit limited to the English language, has not revealed many specific government-led dietary guidelines on vegetarian diets indicating a lack of targeted and available national guidance. Five FBDGs with recommendations or references for vegetarians were identified covering Australia, the Nordic countries in Europe (Iceland, Norway, Denmark, Sweden, Finland and their associated territories, which include the Faroe Islands, Greenland, Svalbard and Åland), Portugal, the Netherlands and the United States (National Health and Medical Research Council, 2013, Nordic Council of Ministers, 2014, Silva et al., 2015; Kromhout et al., 2016, U.S. Department of Health and Human Services and U.S. Department of Agriculture December 2015). The level of detail varies between them as some were specifically developed for vegetarian populations while others are part of more general national dietary guidelines. For instance, the dietary guidelines for a healthy vegetarian population in Portugal is a comprehensive manual which includes considerations for potential nutritional inadequacies and specific guidelines for the general population and school age children (Silva et al., 2015). The dietary guidelines from the Netherlands address vegetarian diets very briefly (Kromhout et al., 2016), while the Australian (National Health and Medical Research Council, 2013) and the Nordic guidelines (Nordic Council of Ministers, 2014) include some nutritional considerations throughout the document. The US dietary guidelines for Americans 2015–2020 provides quantitative recommendations per food group to ensure a healthy vegetarian eating pattern (U.S. Department of Health and Human Services and U.S. Department of Agriculture December 2015).

The documents above usually include valuable considerations to address inadequacies or concerns that have been observed in some vegetarian cohorts (e.g. on protein, n-3 polyunsaturated fatty acids (PUFA), calcium, iron, zinc, iodine, selenium, vitamin B12 and vitamin D). There are also considerations regarding vulnerable population groups with specific nutritional needs including children and pregnant women and importantly for individuals following very restrictive diets. Notwithstanding the focus on critical nutrients in vegetarian diets discussed here, it should be noted that nutrient inadequacies are not limited to vegetarian or vegan diets; large parts of the omnivorous population also have inadequate intakes of several nutrients such as insufficient intakes of iodine, calcium, folic acid and vitamin D or excessive intakes of sodium (Elmadfa et al., 2009; Lazarus, 2014).

#### 2.2.1 Protein

Vegetarians usually meet protein recommendations when adequate energy intake for the individual is achieved. A combination of vegetable proteins such as pulses and whole grains, soy products, nuts, and seeds supplemented with dairy or eggs can provide adequate protein intakes of high biological value. For instance, in the US dietary guidelines for Americans, the derived healthy vegetarian eating pattern recommends soy products, legumes, whole grains, nuts and seeds as well as dairy and eggs to replace meat and fish. For a vegan dietary pattern the diet should include fortified soy or other plant-based milk substitutes (U.S. Department of Health and Human Services and U.S. Department of Agriculture December 2015). However, if only plant protein sources are available, antinutritional factors present in pulses/legumes and whole grains could affect digestibility and bioavailability of protein and amino acids (Boyle et al., 2012). The Italian Society of Human Nutrition suggests vegetarians to increase protein consumption due to the lower digestibility and essential amino acid contents in plant proteins compared to animal proteins. The Portuguese guidelines have adjusted protein intake recommendations for lower digestibility for school age vegan children (3–18 years) (Silva et al., 2015). In addition, it highlights the importance of extending breastfeeding until 2 years of age to ensure the input of high quality protein and other essential nutrients during this growth period.

#### 2.2.2 n-3 PUFA

The consumption of long-chain n-3 PUFA eicosapentaenoic acid (EPA) and docosahexaenoic acid (DHA) are particularly reduced in strict plant-based diets. Vegetarians can improve n-3 PUFA intakes by consuming rich sources of alpha-linolenic acid such as nuts, seeds and their oils (e.g. canola oil) and consuming linoleic acid in moderation. Populations with increased needs for these fatty acids, such as pregnant/breastfeeding women and children, can include dietary sources of long chain PUFA, supplements or fortified foods in the diet.

#### 2.2.3 Vitamin B12

Vitamin B12 is present in small amounts in dairy products but is not supplied by plant foods. The German Nutrition Society states that vitamin B12 is the most critical nutrient on a vegan diet, and the Academy of Nutrition and Dietetics notes that even daily intakes of dairy products as part of an ovo-lacto-vegetarian diet may not be enough to provide the required amount of this vitamin. To achieve adequate intakes, it is important that most vegetarians, and in particular, populations with increased needs such as pregnant, lactating women and vegans, should include reliable sources in their daily diet such as fortified foods (e.g. fortified breakfast cereals and non-animal based milk products) or vitamin B12 supplements (Silva et al., 2015). The Academy of Nutrition and Dietetics points out that vitamin B12 supply from selected food sources is more effective when these foods are ingested on different eating occasions throughout the day, because absorption is easily saturated. The Italian Society of Human Nutrition proposes daily multi-dose and single dose supplementation values for vegetarians based on the adequate intake recommendations of the European Food Safety Authority (EFSA).

#### 2.2.4 Vitamin D

Vitamin D deficiency can affect both vegetarian and non-vegetarian population as most uptake is produced from sun exposure. It is often recommended to vegetarian populations to choose vitamin D rich or fortified food sources to ensure adequate levels of this nutrient (Melina et al., 2016). These can be fortified milk, eggs, fruit juices, breakfast cereals, margarines and mushrooms. Vulnerable populations such as pregnant and breastfeeding women, children, older adults may also consider dietary supplementation for adequate vitamin D intake. This is particularly important when sun exposure is limited and consumption of fortified foods is insufficient to meet nutritional needs.

#### 2.2.5 Calcium

The daily inclusion of dairy products in a vegetarian diet helps meeting calcium recommendations. However, those who completely avoid animal sourced foods should choose efficient plant based calcium sources including green leafy vegetables, nuts, seeds, calcium rich mineral water and calcium fortified foods. The Portuguese guidelines highlight that excess salt consumption may increase calcium excretion in urine (Silva et al., 2015).

#### 2.2.6 Iodine

The inclusion of iodised salt in the diet can provide a good supply of iodine. However, plant based foods can be poor in this micronutrient. Most scientific societies recommend the usage of iodised salt for adequate iodine intakes. The German Nutrition Society and the Academy of Nutrition and Dietetics also state that breastfeeding and lactating women should always take iodine supplements.

#### 2.2.7 Iron

Large variations in iron bioavailability are observed both in omnivorous and vegetarian diets.

Vegetarian populations often consume as much iron as omnivorous but iron body stores, are usually below the normal range (Melina et al., 2016). Haeme-iron from animal products is more efficiently absorbed compared to non-haeme iron from plant sources such as pulses and cereal products. In order to improve iron bioavailability, vegetarians are advised to eat or drink sources of vitamin C such as citrus fruits together with iron rich foods to facilitate absorption and to choose adequate preparation methods such as soaking pulses and whole grains. Iron supplementation can be recommended during pregnancy to prevent iron inadequacy (Melina et al., 2016).

#### 2.2.8 Selenium

Selenium intakes may be reduced among vegetarians but plasma levels usually meet recommendations (Silva et al., 2015; Alles et al., 2017). Selenium levels in eggs, milk, dairy products and plant-based foods are affected by availability of selenium in the soils. The Academy of Nutrition and Dietetics and the Italian Society of Human Nutrition do not refer to this nutrient in their statement, but both the German Nutrition Society and British Dietetic Association advise to include some Brazil nuts in the diet, a particularly rich selenium food source.

#### 2.2.9 Zinc

Compared with non-vegetarians, zinc intake may be reduced among vegetarians (Rizzo et al., 2013; Alles et al., 2017). In addition, zinc absorption can be reduced among vegetarians due to phytates present in plant foods that limit intestinal absorption. However, plant foods such as whole grain provide higher amounts of zinc than refined grains. Nevertheless, there is limited evidence regarding health consequences of low zinc intake in adult vegetarians and populations at risk including pregnant women (Melina et al., 2016). Recommended sources are soy products, beans, whole grains, nuts and seeds as well as zinc-fortified foods such as breakfast cereals. Food preparation techniques such as soaking and sprouting legumes and grains can increase zinc bioavailability (Silva et al., 2015).

#### 2.2.10 Non-nutritive substances

Plant sourced foods contain a broad range of non-nutritive substances including polyphenols, phytoestrogens, phytic acid, and goitrogens. While most of these phytochemicals are associated with physiological effects contributing to the health-promoting effects of plant-sourced foods, some phytochemicals may exert adverse health effects (Watzl and Leitzmann, 2017). Therefore, appropriate food processing technologies have to be applied in order to minimize such adverse effects in vegan/vegetarian diets.

In summary, according to the guidelines and recommendations reviewed, adopting dietary patterns with low environmental footprint, and in particular vegetarian and vegan diets, in a nutritionally adequate and healthy way is feasible but needs attention. There are concerns linked to the exclusion of animal food groups, which are rich in and quality sources of certain essential nutrients. While transitioning to such dietary patterns can provide substantial reductions in the impact food systems exert on the environment and the nitrogen cycle, it is important that such a change remains compatible with good nutrition and health (Westhoek et al., 2014; Godfray et al., 2018; Springmann et al., 2018a).

### 2.3 How to facilitate the adoption of healthy low nitrogen footprint diets

Governments can support healthy food preferences with ‘smart policies' as well depicted by Hawkes et al. (2015). Different actors and networks of producers, retailers, institutions (e.g., schools) and individual citizens can interconnect and act together for a common good. Many policies can affect all parts of such an interconnected network, and food based dietary guidelines are essential bases to governments and actors in ensuring or guiding the shift towards ‘healthy food preferences' (Hawkes et al., 2015).

**Fig. 1:**
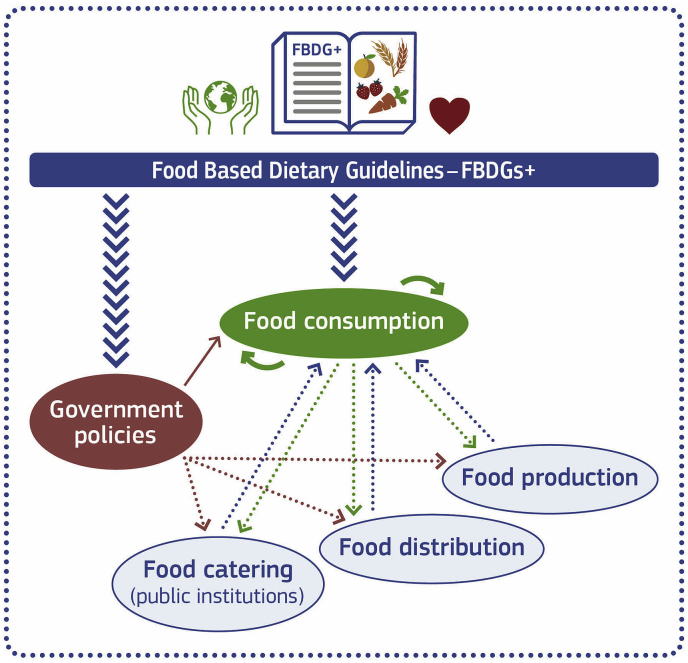
Food based dietary guidelines inclusive of health and sustainability aspects (FBDG+) can influence food consumption. Large blue arrows: FBDG + guide individuals and policies towards consuming and promoting healthy sustainable diets. Red arrows: Smart policies, such as incentives, food standards, legislation or fiscal measures promote healthy low footprint consumer food preferences directly (solid line) or indirectly (dotted line). Green arrows: consumer demand feedbacks to food production, distribution, and catering (dotted line) and peer-influence also nudges other consumers (solid lines) towards healthy low footprint diets. Dotted blue arrows: Food system changes lead to increased availability, ubiquity, and attractiveness of healthy low footprint choices. (For interpretation of the references to color in this figure legend, the reader is referred to the Web version of this article.)

A definition of the principles of how healthy and sustainable diets will be crucial for re-shaping the current food system towards more sustainability. These principles should be co-developed by civil society and governments and inform government-led FBDGs that directly integrate food- and food systems-related environmental sustainability aspects. FBDGs provide dietary guidance to citizens but they are also stepping-stones of nutrition and food-related policy at national level and they can therefore be a vehicle for developing, communicating and implementing a shared view of a sustainable and healthy food system. So far, not many FBDGs include environmental sustainability aspects and developing national FBDGs that also include these considerations is a needed first step to shift towards environmentally friendlier diets (Herforth et al. 2019; Gonzalez Fischer and Garnett, 2016; Bechthold et al., 2018, European Commission, 2019a.). Several nutrition recommendations have recently reconsidered their guidance on protein sources and no longer specify meat consumption, particularly red and processed meat (Herforth et al. 2019, European Commission (2019a.) but whether this is motivated by health or environment reasons or both is not clear. A process needs to be established that allows the development of new food based dietary guidelines – FBDGs + - that are based on the latest science on environmental sustainability and healthy nutrition, possibly including quantitative recommendations for foods and food groups. In high-income countries for example, these FBDGs + will most likely favour the consumption of plant-based foods and suggest a decrease of animal-based foods to achieve meaningful reductions in population's average diet-related environmental footprints, in particular with respect to nitrogen (Blackstone et al., 2018; Willett et al., 2019). They also need to consider specific socio-cultural and socio-economic contexts to ensure they are well accepted by citizens and well-integrated within a national strategy that involves multiple sectors and policy levels (Herforth et al., Keller and Lang, 2008; Gonzalez Fischer and Garnett, 2016). Access to evidence-based guidance that includes health, environmental, socio-cultural and -economic considerations may increase consumer interest in, and acceptability of, predominantly or entirely plant based diets. In turn, shifting social norms would lead to an increased demand for plant-based foods from producers and retailers and reduced meat consumption (Garnett et al., 2015). Fig. 1 builds on health promotion concepts in support of healthy food preferences (Hawkes et al., 2015) and shows a simplified scheme of how FBDGs+ and policies can promote both healthy and sustainable food preferences. Policies such as food procurement standards and economic incentives could increase population's adherence to FBDGs+ and stimulate other actors, such as food producers, retailers and food services to shift their food offer and marketing towards more healthy and sustainable choices (Birt et al., 2017).

Policies to support a more sustainable food system along the chain ‘from farm to fork’ environment are already taking shape in Europe and beyond (European Commission, 2019b; iPES-Food, 2019). While the focus is on increasing resource efficiency and reducing detrimental emissions to the environment during food production and distribution, there are fewer policies that target sustainable food consumption. Nevertheless, recent initiatives at the science-policy interface emphasise the need to stimulate dietary changes towards healthier and more sustainable consumption and to identify solutions that ensure that the food system and its networks converge into favourable and fair societal, economic, environmental and health outcomes (Parsons and Hawkes, 2018, iPES-Food, 2019; SAM, 2019). The resulting shifts in the population demanding more sustainable diets hold multiple benefits to health and well-being, functioning of health care systems, and the environment (Swinburn et al., 2019; Willett et al., 2019). We emphasise here the role of FBDGs and the need to further develop them to include sustainability aspects through a process that is science-based, participatory and inclusive. Such FBDGs + will be a crucial element of, and the reference for, the design of ‘smart’ policies in support of public health and sustainable food systems with low environmental footprint (Fig. 1).

## 3 Conclusion

Given the challenges and the increasing pressure that diet-related burden on both health and environment is imposing on societies, it is urgent to continue striving for health promotion and healthy diets and to include environmental sustainability in the frameworks or recommendations used for this purpose. Human activities are already critically affecting the Earth system's nitrogen cycle and reducing demand for animal-derived dietary protein has been suggested as a key remediation measure (Westhoek et al., 2015; Springmann et al., 2018b; Willett et al., 2019). Nitrogen pollution is among the main factors causing environmental damage from current food systems (Leip and Uwizeye, 2019; Sutton et al., 2011), and it is one of the many examples of failure in meeting sustainability targets (Westhoek et al., 2016; Caron et al., 2018; Béné et al., 2019, iPES-Food, 2019).

The review of the scientific literature presented here shows that even in their most restrictive forms (e.g. vegan, total exclusion of animal-derived products), low environmental footprint diets can be compatible with health goals, although solid trustful information needs to be given to those wishing to follow them. Indeed, there is no direct relationship between a diet's ‘healthiness’ and its sustainability – any diet with low or high environmental footprints (or as here with low or high share of animal-based foods) can be nutritious and healthy provided it is varied and adheres to existing dietary guidelines (Blackstone et al., 2018). More generally, and more encompassing though, we argue that existing FBDGs also need to evolve to guide citizens, decision making and societies towards healthy and lower environmental footprint food preferences. The existing methodological approach to derive FBDGs will need to be adapted and broadened to include sustainability in its wide inclusive definition to help meeting the UN 2030 SDGs. Societies will need to identify suitable means to translate these FBDGs into their national or regional contexts in a more holistic and effective way involving all society sectors and multi-level policies. In addition, different food preferences between individuals will always be present and these dietary guidelines should cater to these differences and include advice for the adoption of healthy low environment footprint, including vegetarian and vegan diets.

## Declaration of competing interest

None.

## References

[bib1] Agnoli C., Baroni L., Bertini I., Ciappellano S., Fabbri A., Papa M., Pellegrini N., Sbarbati R., Scarino M.L., Siani V., Sieri S. (2017). Position paper on vegetarian diets from the working group of the Italian Society of Human Nutrition. Nutr. Metab. Cardiovasc. Dis..

[bib2] Alles B., Baudry J., Mejean C., Touvier M., Peneau S., Hercberg S., Kesse-Guyot E. (2017). Comparison of sociodemographic and nutritional characteristics between self-reported vegetarians, vegans, and meat-eaters from the NutriNet-Sante study. Nutrients.

[bib3] American Dietetic Association and Dietitians of Canada (2003). Position of the American dietetic association and Dietitians of Canada: vegetarian diets. J. Acad. Nutr. Diet..

[bib4] Amit M.S., Paediatric Canadian, Community Paediatrics C. (2010). Vegetarian diets in children and adolescents. Paediatr. Child Health.

[bib5] Barnard N.D., Levin S.M., Yokoyama Y. (2015). A systematic review and meta-analysis of changes in body weight in clinical trials of vegetarian diets. J. Acad. Nutr. Diet..

[bib6] Bechthold A., Boeing H., Tetens I., Schwingshackl L., Nöthlings U. (2018). Perspective: food-based dietary guidelines in Europe—scientific concepts, current status, and perspectives. Adv. Nutr..

[bib7] Béné C., Oosterveer P., Lamotte L., Brouwer I.D., de Haan S., Prager S.D., Talsma E.F., Khoury C.K. (2019). When food systems meet sustainability – current narratives and implications for actions. World Dev..

[bib8] Birt C., Buzeti T., Grosso G., Justesen L., Lachat C., Lafranconi A., Mertanen E., Rangelov N. (2017). Healthy and Sustainable Diets for European Countries.

[bib9] Blackstone N.T., El-Abbadi N.H., McCabe M.S., Griffin T.S., Nelson M.E. (2018). Linking sustainability to the healthy eating patterns of the Dietary Guidelines for Americans: a modelling study. The Lancet Planet. Health.

[bib10] Boyle J., Wijesinha-Bettoni R., Burlingame B. (2012). Protein quality evaluation twenty years after the introduction of the protein digestibility corrected amino acid score method. Br. J. Nutr..

[bib11] Caron P., Ferrero y de Loma-Osorio G., Nabarro D., Hainzelin E., Guillou M., Andersen I., Arnold T., Astralaga M., Beukeboom M., Bickersteth S., Bwalya M., Caballero P., Campbell B.M., Divine N., Fan S., Frick M., Friis A., Gallagher M., Halkin J.-P., Hanson C., Lasbennes F., Ribera T., Rockstrom J., Schuepbach M., Steer A., Tutwiler A., Verburg G. (2018). Food systems for sustainable development: proposals for a profound four-part transformation. Agron. Sustain. Dev..

[bib12] Castañé S., Antón A. (2017). Assessment of the nutritional quality and environmental impact of two food diets: a Mediterranean and a vegan diet. J. Clean. Prod..

[bib13] Corrin T., Papadopoulos A. (2017). Understanding the attitudes and perceptions of vegetarian and plant-based diets to shape future health promotion programs. Appetite.

[bib14] Dagnelie P.C., Mariotti F. (2017). 1 - Vegetarian Diets: Definitions and Pitfalls in Interpreting Literature on Health Effects of Vegetarianism.

[bib15] Dinu M., Abbate R., Gensini G.F., Casini A., Sofi F. (2017). Vegetarian, vegan diets and multiple health outcomes: a systematic review with meta-analysis of observational studies. Crit. Rev. Food Sci. Nutr..

[bib16] Elmadfa I., Meyer A., Nowak V., Hasenegger V., Putz P., Verstraeten R., Remaut-DeWinter A.M., Kolsteren P., Dostalova J. (2009). European nutrition and health report 2009. Forum Nutr..

[bib17] European Commission (2019). Food Based Dietary Guidelines in Europe. https://ec.europa.eu/jrc/en/health-knowledge-gateway/promotion-prevention/nutrition/food-based-dietary-guidelines.

[bib18] European Commission (2019). Reflection Paper towards a Sustainable Europe by 2030.

[bib19] FAO (2018). Nutrient Flows and Associated Environmental Impacts in Livestock Supply Chains Guidelines for Assessment. http://www.fao.org/partnerships/leap/publications/en/.

[bib20] FAO (2019). Food Based Dietary Guidelines. http://www.fao.org/nutrition/education/food-dietary-guidelines/home/en/.

[bib21] Fewtrell M., Bronsky J., Campoy C., Domellöf M., Embleton N., Fidler Mis N., Hojsak I., Hulst J.M., Indrio F., Lapillonne A., Molgaard C. (2017). Complementary feeding: a position paper by the European society for paediatric Gastroenterology, hepatology, and nutrition (ESPGHAN) committee on nutrition. J. Pediatr. Gastroenterol. Nutr..

[bib22] Foster M., Chu A., Petocz P., Samman S. (2013). Effect of vegetarian diets on zinc status: a systematic review and meta-analysis of studies in humans. J. Sci. Food Agric..

[bib23] Galloway J., Winiwarter W., Leip A., Leach A., Bleeker A., JW E. (2014). Nitrogen footprints: past, present and future. Environ. Res. Lett..

[bib24] Garnett T., Mathewson S., Angelides P., Borthwick F. (2015). Policies and Actions to Shift Eating Patterns: what Works.

[bib25] Garton L. (2016, 2 May 2019). Vegetarian diets. https://www.bda.uk.com/foodfacts/vegetarianfoodfacts.pdf.

[bib26] German Federal Ministry of Food and Agriculture (2019). Deutschland, Wie Es Isst - Der BMEL-Ernährungsreport 2019.

[bib27] Godfray H.C.J., Aveyard P., Garnett T., Hall J.W., Key T.J., Lorimer J., Pierrehumbert R.T., Scarborough P., Springmann M., Jebb S.A. (2018). Meat consumption, health, and the environment. Science.

[bib28] Godos J., Bella F., Sciacca S., Galvano F., Grosso G. (2017). Vegetarianism and breast, colorectal and prostate cancer risk: an overview and meta-analysis of cohort studies. J. Hum. Nutr. Diet..

[bib29] Gonzalez Fischer C., Garnett T. (2016). Plates, Pyramids, Planet: Developments in National Healthy and Sustainable Dietary Guidelines: a State of Play Assessment.

[bib30] Haider L.M., Schwingshackl L., Hoffmann G., Ekmekcioglu C. (2018). The effect of vegetarian diets on iron status in adults: a systematic review and meta-analysis. Crit. Rev. Food Sci. Nutr..

[bib31] Hawkes C., Smith T.G., Jewell J., Wardle J., Hammond R.A., Friel S., Thow A.M., Kain J. (2015). Smart food policies for obesity prevention. The Lancet.

[bib32] Herforth, A., M. Arimond, C. Alvarez-Sanchez, J. Coates, K. Christianson and E. Muehlhoff "A Global Review of Food-Based Dietary Guidelines. LID - Nmy130 [pii] LID - 10.1093/advances/nmy130 [doi]." (2156-5376 (Electronic)). 10.1093/advances/nmy130 PMC6628851 31041447

[bib33] Huang R.Y., Huang C.C., Hu F.B., Chavarro J.E. (2016). Vegetarian diets and weight reduction: a meta-analysis of randomized controlled trials. J. Gen. Intern. Med..

[bib34] Huang T., Yang B., Zheng J., Li G., Wahlqvist M.L., Li D. (2012). Cardiovascular disease mortality and cancer incidence in vegetarians: a meta-analysis and systematic review. Ann. Nutr. Metabol..

[bib35] Iguacel I., Miguel-Berges M.L., Gomez-Bruton A., Moreno L.A., Julian C. (2019). Veganism, vegetarianism, bone mineral density, and fracture risk: a systematic review and meta-analysis. Nutr. Rev..

[bib36] iPES-Food (2019). Towards A Common Food Policy for the EU.

[bib37] Janssen M., Busch C., Rödiger M., Hamm U. (2016). Motives of consumers following a vegan diet and their attitudes towards animal agriculture. Appetite.

[bib38] Keller I., Lang T. (2008). Food-based dietary guidelines and implementation: lessons from four countries--Chile, Germany, New Zealand and South Africa. Public Health Nutr..

[bib39] Kromhout D., Spaaij C.J.K., de Goede J., Weggemans R.M. (2016). The 2015 Dutch food-based dietary guidelines. Eur. J. Clin. Nutr..

[bib40] Kwok C.S., Umar S., Myint P.K., Mamas M.A., Loke Y.K. (2014). Vegetarian diet, Seventh Day Adventists and risk of cardiovascular mortality: a systematic review and meta-analysis. Int. J. Cardiol..

[bib41] Lazarus J.H. (2014). Iodine status in europe in 2014. Eur. Thyroid J..

[bib42] Leahy E., Lyons S., Tol R.S.J. (2010). An estimate of the number of vegetarians in the world. Papers WP340, Economic and Social Research Institute (ESRI).

[bib43] Lee Y., Park K. (2017). Adherence to a vegetarian diet and diabetes risk: a systematic review and meta-analysis of observational studies. Nutrients.

[bib44] Leach A.M., Galloway J.N., Bleeker A., Erisman J.W., Kohn R., Kitzes J. (2012). A nitrogen footprint model to help consumers understand their role in nitrogen losses to the environment. Environ. Dev..

[bib45] Leip A., Weiss F., Lesschen J.P., Westhoek H. (2014). The nitrogen footprint of food products in the European Union. J. Agric. Sci..

[bib46] Leip A., Billen G., Garnier J., Grizzetti B., Lassaletta L., Reis S., Simpson D., Sutton M.A., de Vries W., Westhoek H. (2015). Impacts of European livestock production: nitrogen, sulphur, phosphorus and greenhouse gas emissions, land-use, water eutrophication and biodiversity. Environ. Res. Lett..

[bib47] Leip A., Uwizeye A. (2019). Nitrogen footprints. Encycl. Ecol..

[bib48] Leitzmann C., Keller M. (2013). Vegetarische Ernährung. 3..

[bib49] Mekonnen M.M., Hoekstra A.Y. (2012). A global assessment of the water footprint of farm animal products. Ecosystems.

[bib50] Melina V., Craig W., Levin S. (2016). Position of the Academy of nutrition and Dietetics: vegetarian diets. J. Acad. Nutr. Diet..

[bib51] National Health, Medical Research Council (2013). Australian Dietary Guidelines.

[bib52] Nordic Council of Ministers (2014). Nordic Nutrition Recommendations 2012: Integrating Nutrition and Physical Activity.

[bib53] Obersby D., Chappell D.C., Dunnett A., Tsiami A.A. (2013). Plasma total homocysteine status of vegetarians compared with omnivores: a systematic review and meta-analysis. Br. J. Nutr..

[bib54] Parsons K., Hawkes C. (2018). Connecting food systems for co-benefits: how can food systems combine diet-related health with environmental and economic policy goals?. E. O. o. H. S. a. Policies..

[bib55] Pawlak R., Lester S.E., Babatunde T. (2014). The prevalence of cobalamin deficiency among vegetarians assessed by serum vitamin B12: a review of literature. Eur. J. Clin. Nutr..

[bib56] Poore J., Nemecek T. (2018). Reducing food's environmental impacts through producers and consumers. Science.

[bib57] Richter M., Boeing H., Grünewald-Funk D., Heseker H., Kroke A., Leschik-Bonnet E., Oberritter H., Strohm D., Watzl B. (2016). Vegan diet. Position of the German nutrition society (DGE). Ernahrungs umschau.

[bib58] Rizzo N., Jaceldo-Siegl K., Sabate J., Fraser G. (2013). Nutrient profiles of vegetarian and nonvegetarian dietary patterns. J. Acad. Nutr. Diet..

[bib59] SAM (2019). A Scoping Review of Major Works Relevant to Scientific Advice towards an EU Sustainable Food System - Scoping Review Report.

[bib60] Satija A., Bhupathiraju S.N., Spiegelman D., Chiuve S.E., Manson J.E., Willett W., Rexrode K.M., Rimm E.B., Hu F.B. (2017). Healthful and unhealthful plant-based diets and the risk of coronary heart disease in U.S. Adults.. J. Am. Coll. Cardiol..

[bib61] Scarborough P., Appleby P.N., Mizdrak A., Briggs A.D.M., Travis R.C., Bradbury K.E., Key T.J. (2014). Dietary greenhouse gas emissions of meat-eaters, fish-eaters, vegetarians and vegans in the UK. Clim. Change.

[bib62] Segovia-Siapco G., Sabate J. (2018). Health and sustainability outcomes of vegetarian dietary patterns: a revisit of the EPIC-Oxford and the Adventist Health Study-2 cohorts. Eur. J. Clin. Nutr..

[bib63] Silva S., Pinho J., Borges C., Santos C., Santos A., Graça P. (2015). Guidelines for a Healthy Vegetarian Diet. D. G. D. S. National Programme for the Promotion of Healthy Eating.

[bib64] Sobiecki J.G., Appleby P.N., Bradbury K.E., Key T.J. (2016). High compliance with dietary recommendations in a cohort of meat eaters, fish eaters, vegetarians, and vegans: results from the European Prospective Investigation into Cancer and Nutrition–Oxford study. Nutr. Res..

[bib65] Springmann M., Clark M., Mason-D’Croz D., Wiebe K., Bodirsky B.L., Lassaletta L., de Vries W., Vermeulen S.J., Herrero M., Carlson K.M., Jonell M., Troell M., DeClerck F., Gordon L.J., Zurayk R., Scarborough P., Rayner M., Loken B., Fanzo J., Godfray H.C.J., Tilman D., Rockström J., Willett W. (2018). Options for keeping the food system within environmental limits. Nature.

[bib66] Springmann M., Wiebe K., Mason-D'Croz D., Sulser T.B., Rayner M., Scarborough P. (2018). Health and nutritional aspects of sustainable diet strategies and their association with environmental impacts: a global modelling analysis with country-level detail. The Lancet Planet. Health.

[bib67] Steffen W., Richardson K., Rockström J., Cornell S.E., Fetzer I., Bennett E.M., Biggs R., Carpenter S.R., de Vries W., de Wit C.A., Folke C., Gerten D., Heinke J., Mace G.M., Persson L.M., Ramanathan V., Reyers B., Sörlin S. (2015). Planetary boundaries: guiding human development on a changing planet. Science.

[bib68] Sutton M.A., Oenema O., Erisman J.W., Leip A., van Grinsven H., Winiwarter W. (2011). Too much of a good thing. Nature.

[bib69] Swinburn B.A., Kraak V.I., Allender S., Atkins V.J., Baker P.I., Bogard J.R., Brinsden H., Calvillo A., De Schutter O., Devarajan R. (2019). The global syndemic of obesity, undernutrition, and climate change: <em>The lancet</em> commission report. The Lancet.

[bib70] Tilman D., Clark M. (2014). Global diets link environmental sustainability and human health. Nature.

[bib72] U.S. Department of Health and Human Services and U.S. Department of Agriculture (December 2015). 2015 – 2020 Dietary Guidelines for Americans.

[bib73] van Dooren C., Marinussen M., Blonk H., Aiking H., Vellinga P. (2014). Exploring dietary guidelines based on ecological and nutritional values: a comparison of six dietary patterns. Food Policy.

[bib74] Viguiliouk E., Kendall C.W.C., Kahleová H., Rahelić D., Salas-Salvadó J., Choo V.L., Mejia S.B., Stewart S.E., Leiter L.A., Jenkins D.J.A., Sievenpiper J.L. (2018). Effect of vegetarian dietary patterns on cardiometabolic risk factors in diabetes: a systematic review and meta-analysis of randomized controlled trials. Clin. Nutr..

[bib75] Wang F., Zheng J., Yang B., Jiang J., Fu Y., Li D. (2015). Effects of vegetarian diets on blood lipids: a systematic review and meta-analysis of randomized controlled trials. J Am Heart Assoc.

[bib76] Watzl B., Leitzmann C., Mann J., Truswell S.A. (2017). Other biologically active substances in plant foods: phytochemicals. P. 250-260. Essentials of Human Nutrition.

[bib77] Weiss F., Leip A. (2012). Greenhouse gas emissions from the EU livestock sector: a life cycle assessment carried out with the CAPRI model. Agric. Ecosyst. Environ..

[bib78] Westhoek H., Ingram J., Van Berkum S., Özay L., Hajer M. (2016). Food Systems and Natural Resources. A Report of the Working Group on Food Systems of the International Resource Panel.

[bib79] Westhoek H., Lesschen J., Leip A., Rood T., Wagner S., De Marco A., Murphy-Bokern D., Pallière C., Howard C., Oenema O., Sutton M. (2015). Nitrogen on the Table: the influence of food choices on nitrogen emissions and the European environment. European Nitrogen Assessment Special Report on Nitrogen and Food.

[bib80] Westhoek H., Lesschen J.P., Rood T., Wagner S., De Marco A., Murphy-Bokern D., Leip A., van Grinsven H., Sutton M.A., Oenema O. (2014). Food choices, health and environment: effects of cutting Europe's meat and dairy intake. Glob. Environ. Chang..

[bib81] Willett W., Rockström J., Loken B., Springmann M., Lang T., Vermeulen S., Garnett T., Tilman D., DeClerck F., Wood A. (2019). Food in the Anthropocene: the EAT-Lancet Commission on healthy diets from sustainable food systems. The Lancet.

[bib82] Woo K., Kwok T., Celermajer D. (2014). Vegan diet, Subnormal vitamin B-12 status and cardiovascular health. Nutrients.

[bib83] Yokoyama Y., Barnard N.D., Levin S.M., Watanabe M. (2014). Vegetarian diets and glycemic control in diabetes: a systematic review and meta-analysis. Cardiovasc. Diagn. Ther..

[bib84] Yokoyama Y., Levin S.M., Barnard N.D. (2017). Association between plant-based diets and plasma lipids: a systematic review and meta-analysis. Nutr. Rev..

[bib85] Yokoyama Y., Nishimura K., Barnard N.D. (2014). Vegetarian diets and blood pressure: a meta-analysis. JAMA Int. Med..

